# EZH2 coordinates inflammatory and pain-associated pathways across joint compartments in osteoarthritis

**DOI:** 10.1186/s43556-026-00643-w

**Published:** 2026-10-24

**Authors:** Sybille Brochard, Alison Vanlaeys, Mahdia Taieb, Isabelle Richard, Juliette Aury-Landas, Benoit Bernay, Julien Pontin, Celia Seillier, Nina Bon, Baptiste Picart, Claire Pecqueur, Olivier Toutirais, Claire Vinatier, Jérôme Guicheux, Eric Maubert, Véronique Agin, Karim Boumediene, Catherine Bauge

**Affiliations:** 1 UR7451 BIOCONNECT, Université de Caen Normandie, Caen, F-14000 France; 2 US EMerode, PROTEOGEN, Université de Caen Normandie, Caen, 14000 France; 3 Institut Blood and Brain @Caen-Normandie (BB@C), UMR-S U1237, Physiopathology and Imaging of Neurological Disorders (PhIND), Normandie Univ, UNICAEN, INSERM, GIP Cyceron, 14000 Caen, France; 4 Regenerative Medicine and Skeleton (RMeS), UMR 1229, INSERM, Nantes Université, CHU Nantes, Oniris, Nantes, 44000 France; 5 Service de Chirurgie Orthopédique, Hôpital Privé St Martin, 14000 Caen, France; 6 CRCI2NA, CNRS 6075, Inserm 1307, Nantes Université, Université d’Angers, Nantes, F-44000 France

**Keywords:** Osteoarthritis, Pain, Epigenetics, Synoviocytes, Neuroimmune signaling

## Abstract

Enhancer of zeste homolog 2 (EZH2), a histone methyltransferase responsible for H3K27 trimethylation, has emerged as a potential therapeutic target in osteoarthritis (OA). However, its contribution to the multicellular mechanisms driving joint degeneration and pain remains poorly understood. Here, we investigated the effects of pharmacological EZH2 inhibition in a pain-relevant murine OA model and explored its cellular and molecular consequences across OA-associated cell populations. OA was induced in mice by intra-articular monosodium iodoacetate (MIA) injection followed by local administration of the EZH2 inhibitor EPZ-6438. Joint pathology and pain-related behaviors were assessed by histological and functional analyses. Mechanistic studies were performed in primary human OA fibroblast-like synoviocytes and bone marrow-derived cells using targeted gene expression analyses, proteomics and ChIP-seq approaches. EZH2 inhibition reduced cartilage damage, synovial inflammation and pain-related behavioral alterations in vivo. In OA synoviocytes, EPZ-6438 decreased the expression of inflammatory, catabolic and pain-associated mediators while promoting autophagy-related responses. Proteomic and ChIP-seq analyses revealed EZH2-dependent regulation of inflammatory pathways, cellular homeostasis and neuronal-associated processes, including axon guidance-related pathways. ChIP-seq further identified inflammation-dependent EZH2 recruitment to promoters of neurodevelopmental regulators, including PAX6, suggesting a potential contribution of EZH2 to neuronal-associated mechanisms in OA. In addition, EZH2 inhibition reduced macrophage inflammatory activation and osteoclast differentiation. Together, these findings identify EZH2 as a candidate epigenetic regulator linking inflammatory, neuroimmune and osteoimmune pathways across the osteoarthritic joint. Targeting EZH2 may represent a therapeutic strategy to simultaneously modulate joint inflammation, remodeling and pain-associated pathways.

**Supplementary Information:**

The online version contains supplementary material available at https://doi.org/10.1186/s43556-026-00643-w.

## Introduction

Osteoarthritis (OA) is the most prevalent chronic joint disease and a major cause of pain and disability worldwide, affecting more than 500 million individuals globally [1]. Although historically considered a degenerative disorder primarily characterized by cartilage loss, OA is now recognized as a complex whole-joint disease involving dynamic interactions between articular cartilage, synovium, immune cells, subchondral bone, and peripheral sensory nerves [2, 3]. These interactions contribute to both structural deterioration and chronic pain, highlighting the need to identify molecular regulators capable of coordinating pathological responses across multiple joint compartments. Current treatments remain largely symptomatic, and no approved disease-modifying osteoarthritis drug is available to prevent or reverse disease progression [2].

Epigenetic mechanisms have emerged as important contributors to OA pathogenesis by allowing cells to dynamically adapt their transcriptional programs to inflammatory, metabolic, mechanical and aging-related signals [4, 5]. Among these mechanisms, histone H3 lysine 27 trimethylation (H3K27me3), a repressive chromatin mark deposited by the histone methyltransferase Enhancer of Zeste Homolog 2 (EZH2), has attracted particular interest. EZH2 is the catalytic component of the Polycomb Repressive Complex 2 (PRC2), and regulates gene expression through chromatin modification. Several studies, including previous work from our group, have shown that EZH2 contributes to cartilage degeneration by promoting chondrocyte hypertrophy, inflammatory responses, and extracellular matrix degradation [6–9]. However, whether EZH2 also contributes to pathological processes beyond cartilage remains poorly understood.

This question is particularly relevant because OA progression and pain involve multiple joint compartments, including the synovium, immune cells, subchondral bone, and peripheral sensory nerves [10–12]. EZH2 has been implicated in macrophage activation and osteoclast differentiation in other pathological contexts [13, 14], raising the possibility that it may regulate interconnected inflammatory and remodeling processes within the osteoarthritic joint. However, whether EZH2 coordinates these responses across OA-relevant cellular compartments, and whether such effects contribute to pain-associated alterations, remains unknown.

Here, we investigated the role of EZH2 in whole-joint pathology and pain-associated responses in experimental OA. Using pharmacological EZH2 inhibition in the MIA-induced OA model, combined with human OA-derived synoviocytes, macrophages, and osteoclast precursors, we assessed the effects of EZH2 inhibition on joint pathology and cellular responses, and used proteomics and EZH2 ChIP-seq to identify associated molecular pathways and candidate targets. EZH2 inhibition reduced cartilage damage, synovial alterations, and pain-related behavioral changes in vivo, while suppressing inflammatory and catabolic responses in synoviocytes, macrophage activation, and osteoclast differentiation. Together, these findings extend the role of EZH2 beyond cartilage degeneration and identify inflammatory, neuronal-associated, and osteoimmune pathways through which EZH2 may contribute to OA pathogenesis.

## Results

### EZH2 inhibition protects joint integrity and alleviates pain-related alterations in experimental OA

**Fig. 1:**
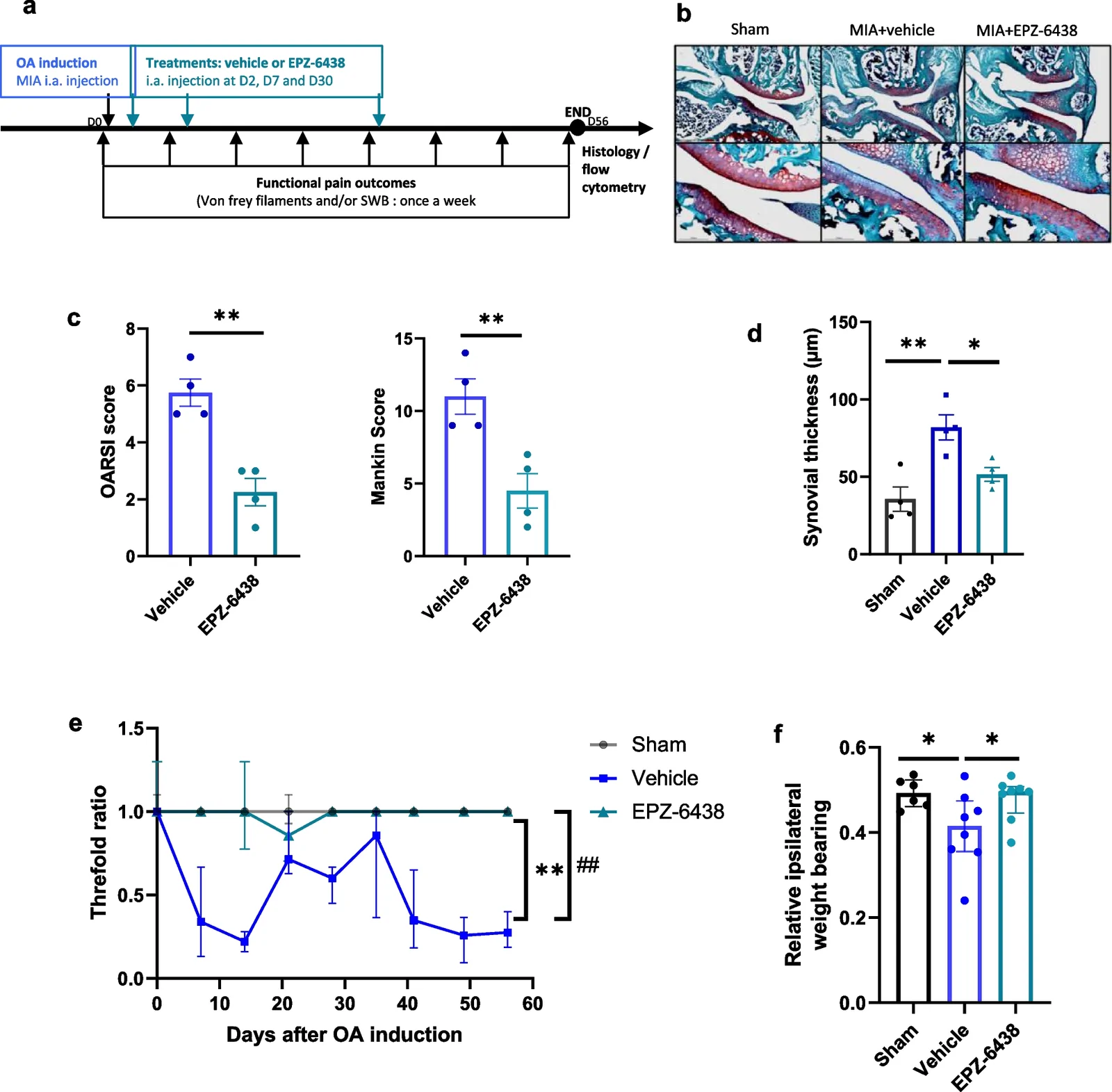
EZH2 inhibition reduces OA progression and pain in a murine model of osteoarthritis. Schematic representation of the experimental design (**a**). Osteoarthritis was induced by intra-articular injection of monosodium iodoacetate (MIA, 0.75 mg diluted in 10 μL of sterile saline solution). Sham animals received intra-articular injection of vehicle. At days 2, 7 and 30 after OA induction, mice received intra-articular injections of the EZH2 inhibitor EPZ-6438 (10 µM, 10 µL) or vehicle into the right knee joint. At day 56, mice were euthanized and histological analyses were performed. Knee joints were processed for Safranin O/Fast Green staining, and representative images were acquired by light microscopy (**b**). Cartilage degradation was evaluated using OARSI and Mankin scores (**c**), and synovial thickness was quantified (**d**). Data are presented as mean ± SEM (n = 4 mice/group). Functional pain was assessed weekly throughout the experiment using von Frey filaments (**e**) and at day 56 using static weight-bearing distribution (**f**). Data are presented as median ± interquartile range (*n* = 8 mice/group). Statistical significance is indicated as *p < 0.05 and p < 0.01. For longitudinal von Frey analysis (**e**), the vehicle-treated OA group was compared with the sham group (##) and the EPZ-6438-treated OA group (**)

Previous studies have established EZH2 as a regulator of cartilage degeneration in surgically induced OA models, but its contribution to OA-associated pain remains unclear. We therefore used the monosodium iodoacetate (MIA)-induced OA model, which combines progressive joint structural damage with robust pain-related behavioral alterations. Following intra-articular MIA injection, mice received local administration of the selective EZH2 inhibitor EPZ-6438 at days 2, 7, and 30 after OA induction (Fig. 1a). MIA-treated mice developed marked cartilage degradation at day 56, whereas EPZ-6438-treated animals showed significantly lower cartilage damage scores (Fig. 1b-c). Consistent with these findings, synovial membrane thickening was reduced following EZH2 inhibition (Fig. 1d). Functional assessment further showed that EPZ-6438 reduced mechanical hypersensitivity and partially restored weight distribution on the affected limb (Fig. 1e–f). Together, these findings indicate that local EZH2 inhibition attenuates both structural joint damage and pain-related alterations in experimental OA.

### EZH2 drives inflammatory and catabolic responses in OA synoviocytes

**Fig. 2:**
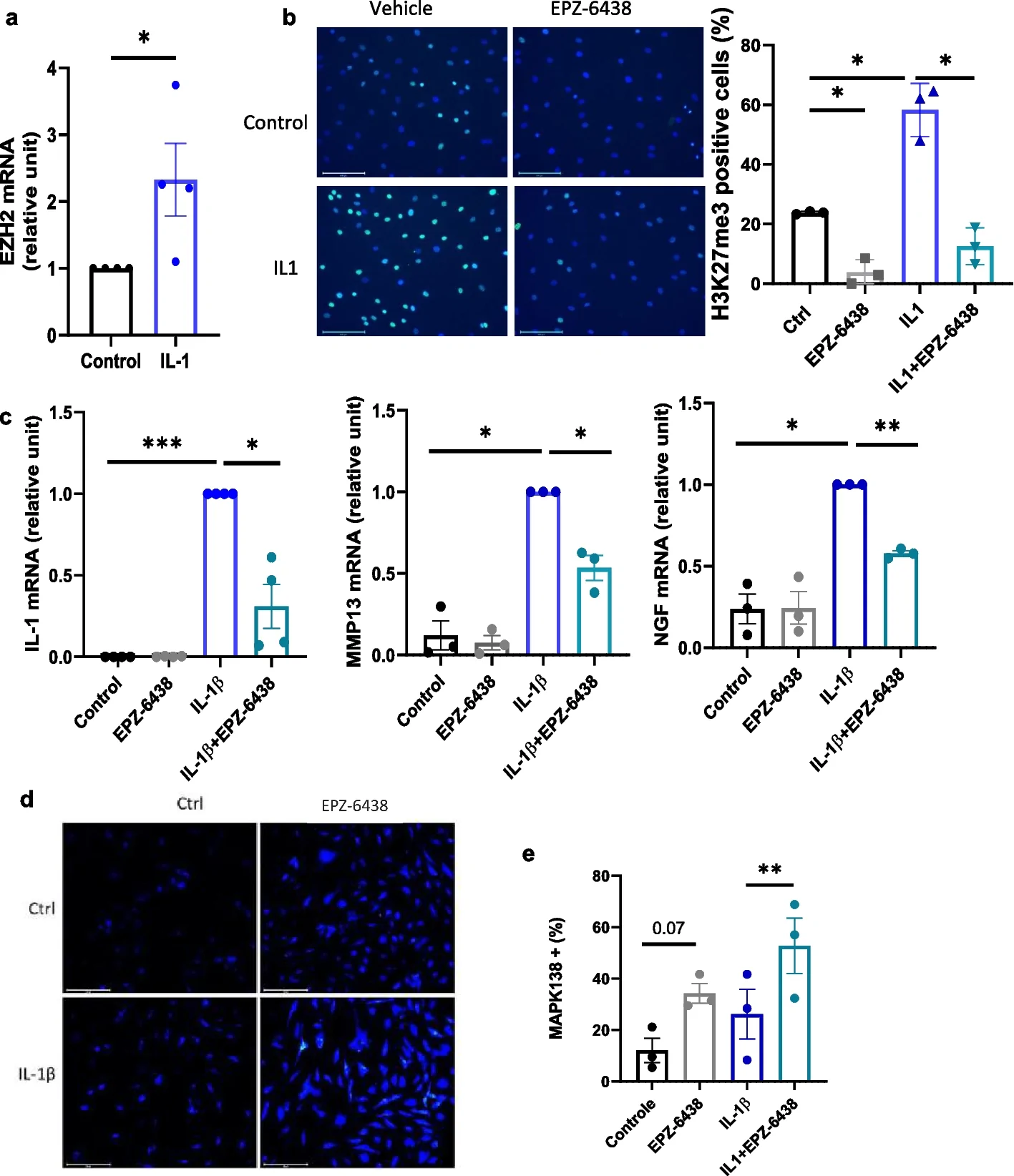
EZH2 inhibition modulates inflammatory, catabolic, pain-related and autophagic responses in fibroblast-like synoviocytes. Human fibroblast-like synoviocytes were stimulated for 48 h with IL-1β (1 ng/mL). EZH2 mRNA expression was analyzed by RT-qPCR (**a**). Cells stimulated with IL-1β in the presence or absence of EPZ-6438 (10 µM) were stained for H3K27me3 (FITC, green) by immunocytochemistry, and nuclei were counterstained with DAPI. Representative images from three independent experiments are shown. Quantification of H3K27me3-positive cells is presented (**b**). The expression of inflammation-, catabolism- and pain-related genes (IL1B, MMP13 and NGF) was analyzed by RT-qPCR (**c**). Autophagy-associated signal was assessed using a fluorescent autophagosome detection assay (MAK138). Representative images from three independent experiments are shown (d). The MAK138-positive area was quantified and expressed as a percentage of total cell area (**e**). Data are presented as mean ± SEM from at least three independent biological experiments. Statistical significance is indicated as *p < 0.05, **p < 0.01 and ***p < 0.001

Because synovial inflammation contributes substantially to OA progression and pain sensitization, we next investigated whether synovial fibroblasts represent a cellular target of EZH2 activity. Human OA-derived fibroblast-like synoviocytes (FLS) were stimulated with IL-1β to reproduce an inflammatory OA-like environment. As shown in Fig. 2a, IL-1β stimulation increased EZH2 expression in OA-FLS. This was accompanied by an increase in H3K27me3 staining, whereas EPZ-6438 reduced H3K27me3 accumulation (Fig. 2b), confirming inhibition of EZH2 enzymatic activity. EPZ-6438 further reduced the IL-1β-induced expression of IL1B, MMP13, and NGF (Fig. 2c). In parallel, increased autophagosome was observed following EZH2 inhibition (Fig. 2d-e). These results identify OA synoviocytes as an EZH2-responsive cellular compartment and indicate that EZH2 inhibition suppresses inflammatory, catabolic and pain-associated responses while promoting autophagy-associated cellular processes.

### Proteomic profiling identifies inflammatory, metabolic and neuronal-associated pathways regulated by EZH2 inhibition

To characterize the molecular consequences of EZH2 inhibition in inflammatory synoviocytes, we performed quantitative proteomic profiling of IL-1β-stimulated OA-derived FLS treated with EPZ-6438.

**Fig. 3:**
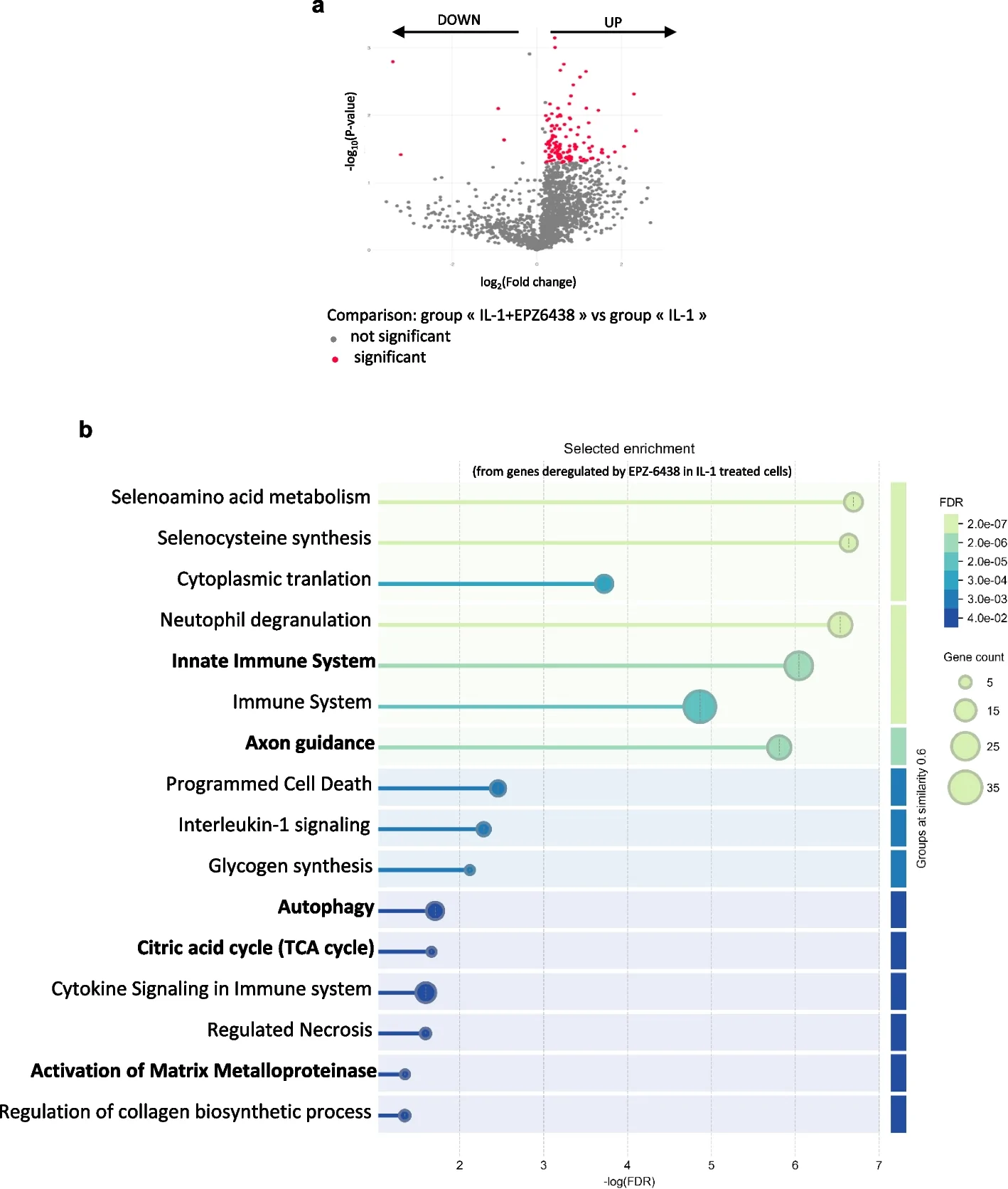
Proteomic profiling identifies inflammatory, metabolic and neuronal-associated pathways regulated by EZH2 inhibition. Human fibroblast-like synoviocytes from OA patients (n = 3 independent biological replicates) were stimulated for 48 with IL-1β (1 ng/mL) in the presence or absence of EPZ-6438 (10 µM). Total proteins were extracted and analyzed by label-free quantitative proteomics. Differentially abundant proteins between IL-1β and IL-1β + EPZ-6438 conditions were identified using a volcano plot representation (**a**). Proteins displaying a fold change ≥ 1.2 and a *p*-value < 0.05 were considered differentially abundant. Gene Ontology Biological Process and Reactome pathway enrichment analyses were performed using STRING database (**b**)

This analysis identified 128 proteins whose abundance was significantly altered by EPZ-6438 treatment (p < 0.05; fold change > 1.2), with the majority showing increased abundance following EZH2 inhibition (Fig. 3a; Table S1). Functional enrichment analysis showed that these proteins were enriched in pathways related to inflammatory signaling, extracellular matrix regulation, and autophagy (Fig. 3b). Notably, neuronal-associated pathways, particularly axon guidance, were also significantly enriched. Metabolic pathways, including the TCA cycle and glycogen metabolism, were additionally identified among the regulated processes. Together, these findings indicate that EZH2 inhibition induces broad molecular changes in inflammatory synoviocytes, involving inflammatory, metabolic, cellular homeostasis, and neuronal-associated pathways.

### EZH2 inhibition modulates representative metabolic and neuronal genes in inflammatory synoviocytes

**Fig. 4:**
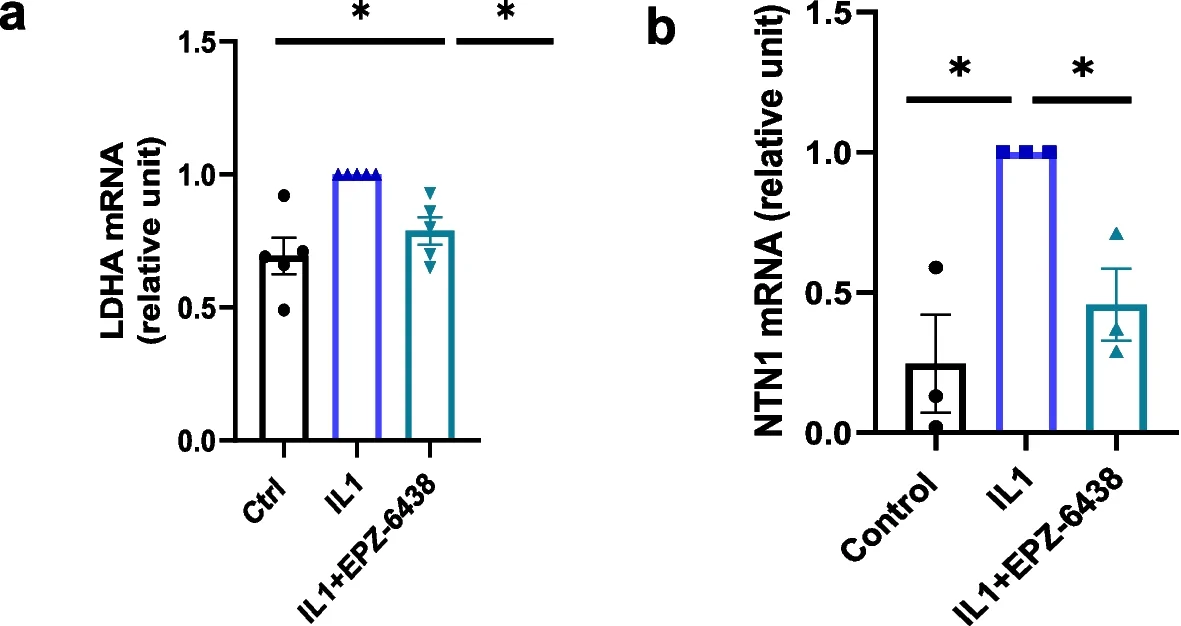
EZH2 inhibition modulates representative metabolic and neuronal genes in inflammatory synoviocytes. Human fibroblast-like synoviocytes were stimulated for 48 h with IL-1β (1 ng/mL) in the presence or absence of EPZ-6438 (10 µM). RNA was extracted and LDHA (**a**) and NTN1 (**b**) mRNA expression was analyzed by RT-qPCR. Data are presented as mean ± SEM from at least three independent OA patients. Statistical significance is indicated as *p < 0.05

Because proteomic analysis identified several metabolism-related pathways, we first examined LDHA expression, which was significantly reduced by EPZ-6438 (Fig. 4a). However, Seahorse analysis did not reveal significant alterations in glycolytic or mitochondrial function (Fig. S1), suggesting that EZH2 inhibition modifies metabolic gene expression without inducing major bioenergetic remodeling. We next investigated the neuronal guidance molecule NTN1, which was selected because of its reported role in sensory nerve ingrowth and OA pain. IL-1β increased NTN1 expression, whereas EPZ-6438 markedly reduced this induction (Fig. 4b). These findings identify NTN1 as an EZH2-responsive neuro-associated factor in inflammatory synoviocytes and provide a potential molecular link between synovial inflammation and pathways involved in sensory nerve regulation.

### ChIP-seq identifies neurodevelopmental genes as potential EZH2 targets in inflammatory synoviocytes

**Fig. 5:**
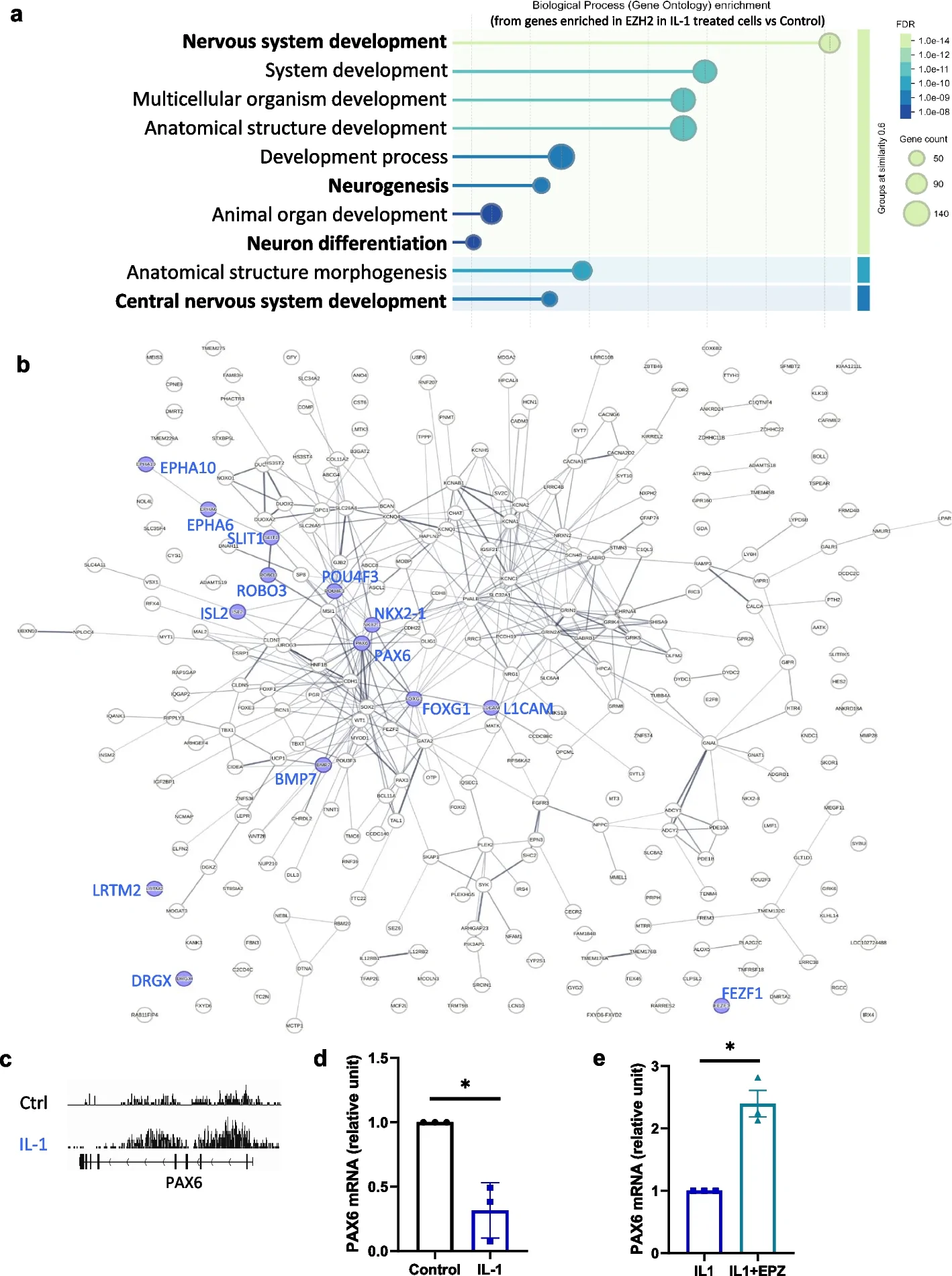
ChIP-seq identifies neurodevelopmental genes as potential EZH2 targets in inflammatory synoviocytes. Fibroblast-like synoviocytes were incubated for 48 h with IL-1β (1 ng/ml), and EZH2 genomic occupancy was investigated by ChIP-seq using an EZH2-specific antibody. Sequencing data were analyzed by identifying common and condition-specific EZH2 peaks. Functional enrichment analysis (Gene Ontology Biological Processes) was performed on genes associated with EZH2 promoter peaks specifically identified under IL-1β stimulation using STRING database (**a**). The protein–protein interaction network of genes associated with IL-1β-specific EZH2 peaks is shown, with genes related to the "axon guidance" pathway highlighted in blue (**b**). Representative ChIP-seq peaks corresponding to the PAX6 locus are shown (**c**). PAX6 mRNA expression was analyzed by RT-PCR (**d**), and the effect of EPZ-6438 treatment on PAX6 expression was assessed (**e**). ChIP-seq experiments were performed using biological replicates from two independent OA patients. RT-PCR experiments were independently performed using biological replicates from three different OA patients Data are presented as mean ± SEM. *: *p*-value < 0.05

To further investigate potential epigenetic mechanisms underlying these responses, genome-wide EZH2 ChIP-seq analysis was performed in IL-1β-stimulated FLS. Inflammatory stimulation induced the recruitment of EZH2 to promoter regions corresponding to 285 genes. Functional enrichment analysis revealed a significant association with nervous system development and axon guidance pathways, including genes involved in neuronal differentiation and developmental regulation (Fig. 5a-b and S2). Among these candidates, PAX6 was selected for further validation because of its established role in neuronal development and axonal guidance. EZH2 enrichment was detected at the PAX6 locus (Fig. 5c), and inflammatory stimulation reduced PAX6 expression, whereas EZH2 inhibition restored PAX6 expression levels (Fig. 5d-e). Together, these data identify neurodevelopmental genes as candidate EZH2 targets in inflammatory synoviocytes, providing candidate molecular links between epigenetic regulation and neuronal-associated pathways in OA.

### EZH2 inhibition reduces inflammatory macrophage activation

**Fig. 6:**
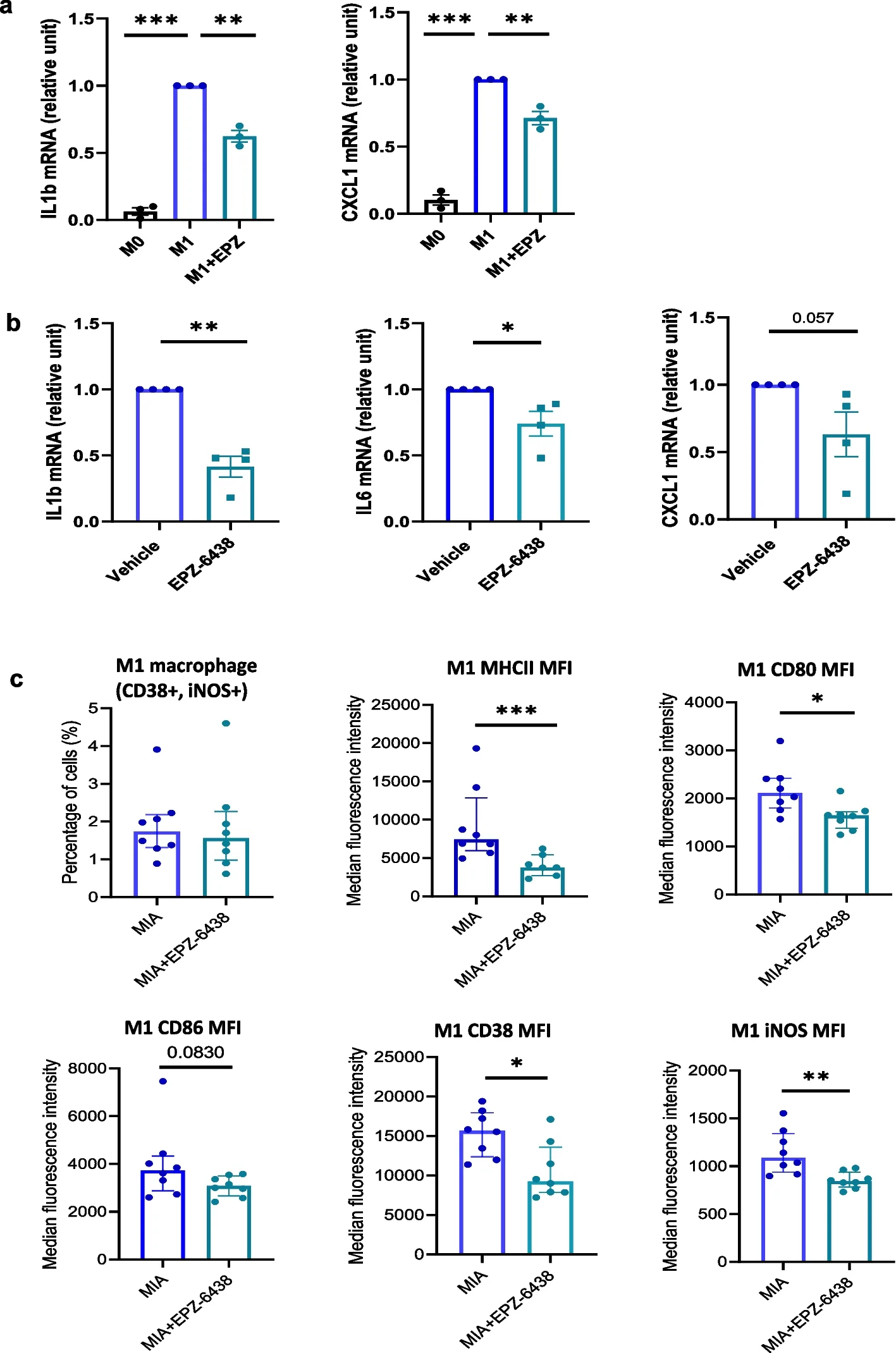
EZH2 inhibition reduced the inflammatory phenotype of macrophages during OA. Macrophage differentiation and M1-like polarization were induced from the THP-1 monocytic cell line (**a**) or from human bone marrow-derived cells isolated from OA patients (**b**) in the presence or absence of EPZ-6438. The expression of pro-inflammatory markers (IL-1β, IL-6 and CXCL1) was analyzed by RT-qPCR. Experiments using THP-1 cells were independently repeated at least three times. Experiments using human bone marrow-derived cells were performed using biological replicates from independent OA patients. Data are presented as mean ± SEM. *: *p*-value < 0.05; **: *p*-value < 0.01; ***: *p*-value < 0.001. Spleens from OA mice described in Fig. 1 and treated with vehicle or EPZ-6438 were collected and analyzed by flow cytometry. Cells were stained for macrophage-associated markers (**c**). Samples were acquired using a FACSVerse flow cytometer and analyzed with FlowJo 7.6.5 software. Results are presented as median ± interquartile range (n = 8 mice). *: *p*-value < 0.05; **: *p*-value < 0.01

Because macrophages are major regulators of synovial inflammation and OA-associated pain, we next examined whether EZH2 also controls macrophage activation. THP-1-derived macrophages polarized toward an inflammatory M1-like phenotype in the presence of EPZ-6438 displayed reduced expression of inflammatory mediators, including IL-1β and CXCL1 (Fig. 6a). Similar effects were observed in macrophages differentiated from OA patient-derived bone marrow cells, where EZH2 inhibition decreased IL-1β, IL-6, and CXCL1 expression (Fig. 6b). To determine whether EZH2 inhibition influenced immune responses in vivo, immune cells from OA mice treated with EPZ-6438 were analyzed by flow cytometry. EPZ-6438-treated animals displayed reduced expression of inflammatory macrophage-associated markers, including MHCII, CD80, CD38, and iNOS (Fig. 6c and S3). Together, these findings demonstrate that EZH2 inhibition attenuates inflammatory macrophage activation both in human cell models and in the OA joint in vivo.

### EZH2 inhibition suppresses osteoclast differentiation

**Fig. 7:**
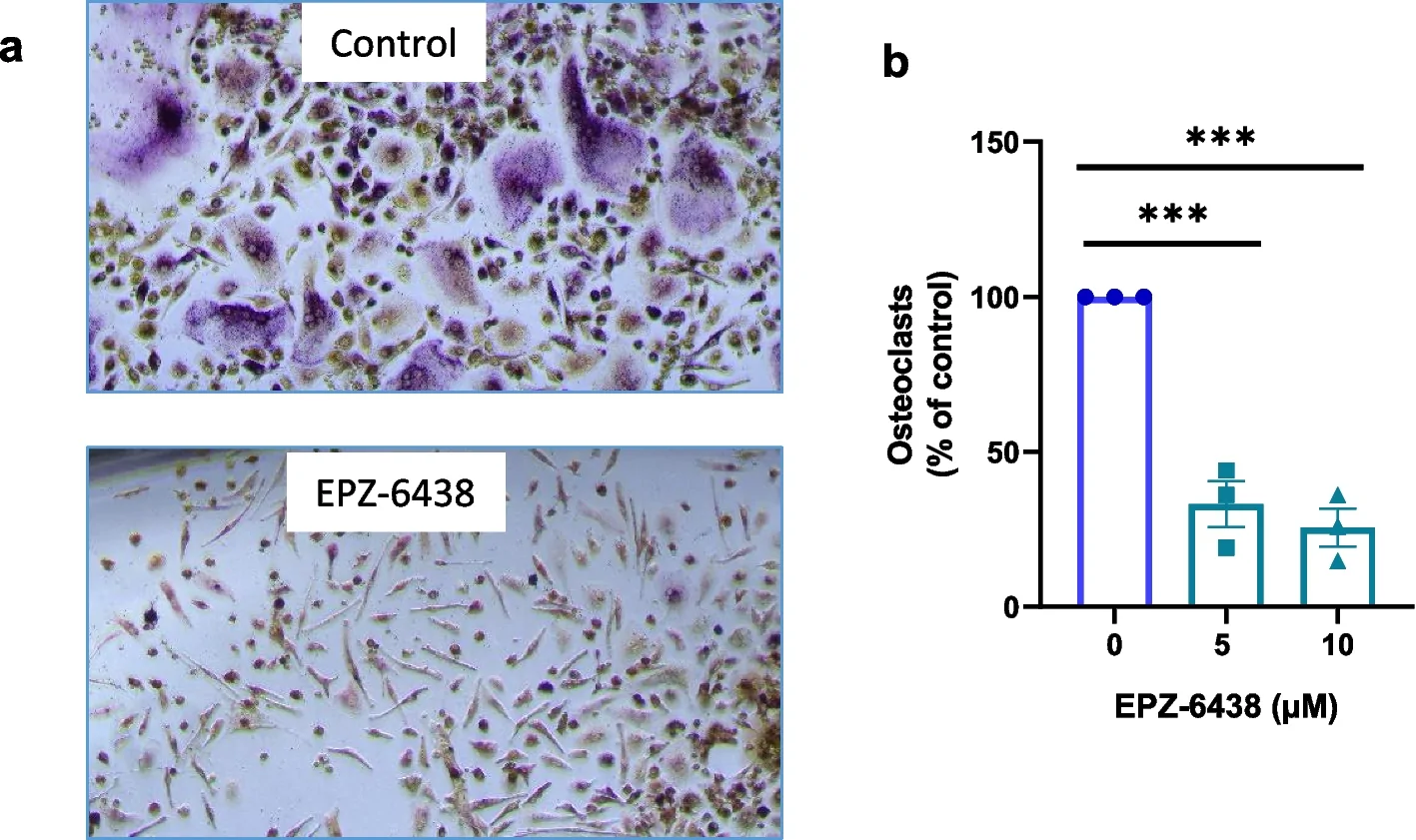
EZH2 inhibition reduced osteoclastogenesis. Bone marrow-derived cells were isolated from OA patients. After ACK lysis, cells were cultured at 300,000 cells/cm.^2^ in α-MEM supplemented with 10% FBS, penicillin–streptomycin, 50 µM β-mercaptoethanol and osteoclastogenic cytokines (RANK-L, 30 ng/mL; M-CSF, 25 ng/mL) in the presence or absence of EPZ-6438 (5 or 10 µM). When multinucleated giant cells were observed (approximately seven days), osteoclast differentiation was assessed by TRAP staining according to the manufacturer's protocol. Representative images are shown (**a**), and TRAP-positive multinucleated osteoclasts were counted and expressed as a percentage of the untreated control condition (set at 100%) (**b**). Results are presented as mean ± SEM from three independent OA patients (*n* = 3). ***: *p*-value < 0.001

Subchondral bone remodeling represents another important component of OA progression and pain. Since EZH2 has previously been implicated in osteoclast differentiation, we investigated whether EZH2 inhibition affects osteoclastogenesis in OA-derived cells. Bone marrow-derived progenitors isolated from OA patients were induced to differentiate into osteoclasts in the presence or absence of EPZ-6438. EZH2 inhibition significantly reduced formation of TRAP-positive multinucleated osteoclasts (Fig. 7a-b). Together, these data indicate that EZH2 contributes to osteoclastogenic responses in OA-derived bone marrow cells, extending the effects of EZH2 inhibition to the osteoimmune compartment.

## Discussion

Osteoarthritis is increasingly recognized as a whole-joint disease in which reciprocal interactions between cartilage, synovium, immune cells, subchondral bone and sensory nerves collectively drive structural damage and chronic pain. This evolving concept challenges the traditional cartilage-centered view of OA and highlights the need to identify upstream regulators capable of coordinating pathogenic responses across multiple joint compartments. In the present study, we demonstrate that pharmacological inhibition of EZH2 attenuates structural joint damage and pain-related behaviors while simultaneously modulating inflammatory, neuronal-associated and osteoimmune pathways in several OA-relevant cell populations. Together, our findings extend the role of EZH2 beyond cartilage degeneration and support a broader role for EZH2 in coordinating multiple OA-relevant cellular responses.

**Fig. 8:**
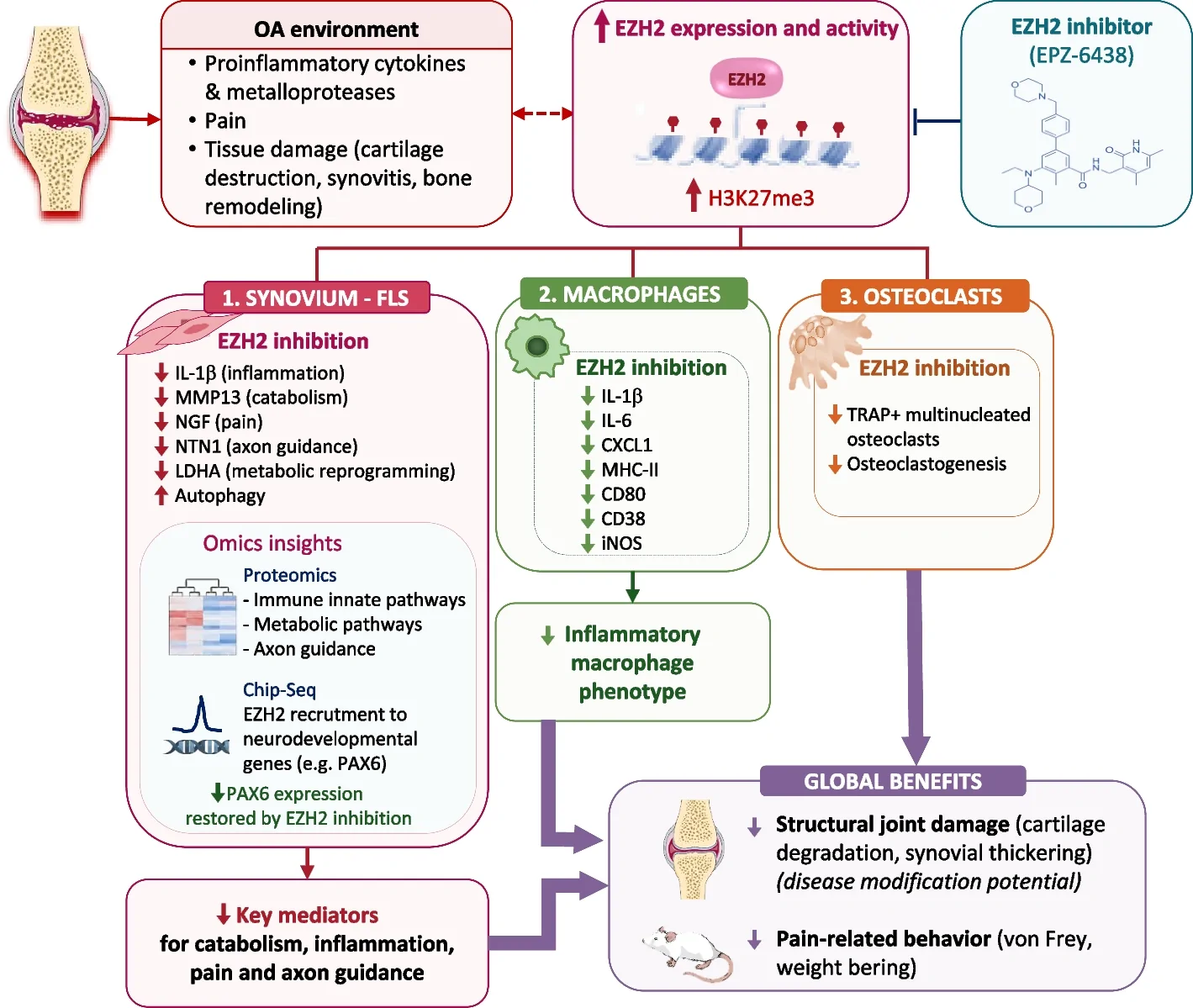
Proposed model illustrating the effects of EZH2 inhibition across multiple joint compartments in osteoarthritis. In OA fibroblast-like synoviocytes (FLS), increased EZH2 and H3K27me3 levels were associated with inflammatory and catabolic responses, which were reduced by pharmacological EZH2 inhibition with EPZ-6438. In human OA FLS, EPZ-6438 decreased inflammatory, catabolic and pain-associated mediators, including IL1B, MMP13, NGF and NTN1, reduced LDHA expression and increased autophagy-associated responses. Proteomic and ChIP-seq analyses further identified inflammatory, metabolic and axon guidance-related pathways, including inflammation-dependent EZH2 recruitment at the PAX6 locus. EZH2 inhibition also reduced inflammatory activation of macrophages and osteoclastogenesis in OA-derived bone marrow cells. Collectively, these findings support a broader role for EZH2 in inflammatory, catabolic and osteoimmune responses across multiple OA-relevant cellular compartments. The identification of PAX6, NTN1 and axon guidance-related pathways highlights neuro-associated molecular programs that may be relevant to pain-associated responses, although their direct contribution to pain or sensory nerve remodeling was not assessed in this study. The reduced structural joint damage and pain-related behaviors observed following EZH2 inhibition in the MIA-induced OA model are consistent with this multicellular framework

Previous studies, including our own, have demonstrated that EZH2 promotes cartilage degeneration by enhancing chondrocyte hypertrophy, inflammatory responses and extracellular matrix degradation [6–9]. Our data considerably broaden this view by showing that EZH2 activity is not restricted to cartilage but is also engaged in synovial fibroblasts, inflammatory macrophages and osteoclast precursors. This broader activity is consistent with the emerging concept that epigenetic regulators function as integrators of environmental signals, enabling coordinated transcriptional responses to inflammatory, mechanical and metabolic stress. Rather than acting within a single tissue, EZH2 therefore appears to orchestrate pathological responses across several cellular compartments that collectively contribute to OA progression (Fig. 8).

A major finding of this study is the identification of fibroblast-like synoviocytes as an important EZH2-responsive compartment. Synovial fibroblasts are increasingly recognized as active drivers of OA progression through the production of inflammatory cytokines, matrix-degrading enzymes and soluble mediators capable of influencing immune cell recruitment and nociceptive signaling. In our study, inflammatory stimulation increased both EZH2 expression and H3K27me3 accumulation, while pharmacological inhibition reduced IL1B, MMP13 and NGF expression and enhanced autophagy. These observations indicate that EZH2 contributes to the establishment of a pathogenic synovial phenotype and suggest that restoration of cellular homeostasis through enhanced autophagy may participate in the protective effects of EZH2 inhibition. Together with recent transcriptomic studies demonstrating extensive inflammatory and stromal remodeling within OA synovium [15], our findings reinforce the concept that synovial fibroblasts constitute a major therapeutic target for epigenetic intervention.

One of the most intriguing observations of this work is the identification of neuronal-associated pathways among the principal molecular programs regulated by EZH2. Although pain is the predominant clinical manifestation of OA, the molecular mechanisms linking synovial inflammation to sensory nerve remodeling remain incompletely understood. Our proteomic analyses unexpectedly identified axon guidance among the most significantly enriched pathways following EZH2 inhibition. Consistent with these observations, EPZ-6438 reduced inflammatory induction of NTN1, a guidance molecule previously implicated in sensory nerve sprouting and pain sensitization during OA [16, 17]. Furthermore, genome-wide ChIP-seq identified inflammation-dependent recruitment of EZH2 to promoters of genes involved in nervous system development, including PAX6, whose expression was restored after EZH2 inhibition. Although these findings do not establish a direct causal role for PAX6 in OA pathogenesis, they identify neurodevelopmental genes as previously unrecognized EZH2-associated targets in inflammatory synoviocytes. More broadly, they suggest that epigenetic regulation within stromal cells may contribute to neuroimmune communication in the osteoarthritic joint, thereby providing one potential explanation for the reduction in pain-related behaviors observed after EZH2 inhibition.

Beyond resident synovial cells, our study demonstrates that EZH2 also regulates inflammatory and remodeling responses in immune and skeletal compartments. EPZ-6438 reduced inflammatory activation of macrophages both in vitro and in vivo, consistent with previous studies identifying EZH2 as a regulator of macrophage polarization [13, 18]. Because activated macrophages contribute to synovitis, cytokine production and peripheral sensitization [12], modulation of macrophage phenotype is likely to participate in the overall therapeutic effects observed in vivo. Likewise, inhibition of osteoclast differentiation extends previous reports describing EZH2 as a positive regulator of osteoclastogenesis [14, 19]. Since subchondral osteoclast activity promotes bone remodeling, angiogenesis and sensory nerve ingrowth during OA progression [20], these findings are consistent with a role of EZH2 in regulating osteoimmune mechanisms contributing both to structural damage and pain generation.

Interestingly, quantitative proteomics also identified several metabolic pathways among proteins regulated by EZH2 inhibition. However, despite reduced expression of LDHA, complementary Seahorse analyses did not reveal major alterations in glycolytic or mitochondrial activity. These results suggest that the metabolic signatures identified by proteomics more likely reflect secondary adaptations accompanying the anti-inflammatory response than a primary metabolic reprogramming induced by EZH2 inhibition. Future studies combining metabolic flux analyses with longitudinal kinetic assessments will be important to define the temporal dynamics of metabolic responses to EZH2 inhibition.

Several limitations should nevertheless be acknowledged. First, the present study relies primarily on pharmacological inhibition of EZH2 using EPZ-6438. Although this clinically approved inhibitor offers strong translational relevance, cell-specific genetic approaches will be required to determine the relative contribution of individual joint compartments to the therapeutic effects observed in vivo. Second, while the MIA model faithfully reproduces pain-related alterations and several pathological features of OA, it does not encompass the full complexity of human disease. Confirmation of these findings in additional experimental models will therefore be important. Finally, although our study identifies candidate neuroimmune pathways involving NTN1 and PAX6, their precise functional contribution to sensory nerve remodeling and OA pain remains to be experimentally demonstrated.

In conclusion, our findings identify EZH2 as a central epigenetic regulator associated with inflammatory, neuroimmune and osteoimmune pathways in OA. Rather than acting exclusively on cartilage, EZH2 appears to integrate pathogenic mechanisms operating within stromal, immune and skeletal compartments, ultimately influencing both structural progression and pain. These results provide a conceptual framework supporting local EZH2 inhibition as a promising strategy to simultaneously target multiple disease-driving mechanisms in osteoarthritis.

## Material and methods

### Reagents

For EZH2 inhibition experiments, EPZ-6438 (tazemetostat; Selleckchem, Souffelweyersheim, France) was dissolved in dimethyl sulfoxide (DMSO; Dutscher, Bernolsheim, France) at a stock concentration of 10 mM and stored at −20 °C until use. For in vitro experiments, EPZ-6438 was diluted directly into culture medium to obtain a final concentration of 10 µM. For in vivo experiments, EPZ-6438 was diluted in sterile saline solution to obtain a final concentration of 10 µM for intra-articular injection. Human recombinant IL-1β (SRP3083, Sigma-Aldrich, St Louis, USA) was reconstituted in DPBS supplemented with 0.1% BSA at 1 µg/mL and used at a final concentration of 1 ng/mL. Phorbol 12-myristate 13-acetate (PMA; MedChemExpress, Monmouth Junction, USA) was dissolved in DMSO at a stock concentration of 20 mM. Recombinant human interferon gamma (IFNγ; PreproTech, Cranbury, USA) was prepared in DPBS containing 0.1% BSA to a final concentration of 10 µg/mL.

### Animals

The in vivo experiments included 27 male C57BL/6 J mice, 8 weeks of age, obtained from Janvier Labs (France). Male mice were selected as they represent the most commonly used sex in monoiodoacetate (MIA)-induced osteoarthritis models and allow comparison with previous studies investigating EZH2 inhibition in experimental OA. Mice were maintained at the Centre Universitaire de Ressources Biologiques (CURB, University of Caen Normandy, France) under controlled environmental conditions, including a temperature of 23 ± 2 °C and a 12-h reversed light/dark cycle. Food and water were available ad libitum. Animal procedures were performed between 8:00 AM and 5:00 PM under dim illumination (6 lx).

All experimental procedures complied with the European Directive 2010/63/EU and French legislation on animal experimentation (decree 87–848). The study was conducted at the GIP CYCERON/CURB facility (approval #D14118001) and was approved by the French Ministry of Education and Research and the regional ethics committee CENOMEXA (APAFIS number #16,185). Experimental procedures were performed according to ARRIVE guidelines. Every effort was made to minimize animal suffering and reduce the number of animals used.

Animals were randomly allocated to the experimental groups. A subset of animals was used for behavioral and histological analyses, whereas spleens and lymph nodes from treated animals were collected for flow cytometry analyses.

### Induction of experimental osteoarthritis and treatment

The experiments were performed according the timeline presented in Fig. 1a. Experimental osteoarthritis was induced by intra-articular injection of monosodium iodoacetate (MIA; Sigma-Aldrich) as previously described [21].On day 0, experimental OA was induced by administering 0.75 mg MIA in 10 µL of sterile saline into the right knee joint. For the sham procedure, the same volume of sterile saline was injected intra-articularly into the right knee. EPZ-6438 treatment was administered intra-articularly into the right knee joint at days 2, 7, and 30 after OA induction. EPZ-6438 was diluted from a 10 mM stock solution prepared in DMSO to a final concentration of 10 µM in sterile saline solution (10 µL/injection, corresponding to 0.043 µg EPZ-6438 per injection). Control OA animals received intra-articular injections of vehicle at the same time points. Animals were euthanized at day 56 after OA induction by cervical dislocation under deep anesthesia (5% isoflurane, 70% N₂O/30% O₂).

### Histological analysis and cartilage scoring

At day 56, knee joints were collected and fixed in 10% formalin (4% formaldehyde) for 48 h. Samples were subsequently decalcified in neutral 10% EDTA/PBS for 5 days, cryoprotected in 30% sucrose solution for 48 h, embedded in OCT compound, and stored at − 80°C. Sections (10 µm thickness) were obtained using a cryostat (CM3050S, Leica Biosystems, Nanterre, France), mounted on Superfrost Plus slides (Thermo Fisher Scientific), and stored at − 80 °C until staining. Safranin O staining was performed, followed by counterstaining with Fast Green as previously described [21]. Cartilage degradation was assessed using the OARSI scoring system adapted for murine osteoarthritis [22] and the Mankin score [23]. Synovial alterations were evaluated by measuring synovial membrane thickness. Images were acquired using a Leica DM6 microscope equipped with a DMC2900 camera.

### Functional pain assessment

OA-associated pain behavior was assessed longitudinally using von Frey filament testing and at the end of the experiment using static weight-bearing (SWB) analysis. Before behavioral experiments, mice underwent a three-week habituation period with regular handling twice weekly to reduce stress associated with manipulation. Behavioral procedures were performed at the HANDIFORM platform (CYCERON/CURB, Caen, France).

Mechanical tactile sensitivity was evaluated once a week after OA induction using von Frey filaments (Ugo Basile, Salon-de-Provence, France), as previously described [21]. Mice were placed individually in elevated grid cages and allowed to acclimatize for 1 h before testing. Mechanical stimulation was applied to the plantar surface of each hind paw using calibrated nylon filaments. A positive response was defined as paw withdrawal, snapping, or licking following stimulation. Three positive responses among a maximum of five stimulations were considered indicative of a response threshold. Results were expressed as the ratio between withdrawal responses of the OA-injected right paw and the contralateral left paw.

Static weight-bearing distribution was assessed once at day 56 using a Bioseb SWB system (Vitrolles, France). After one day of habituation to the apparatus, ten consecutive measurements were performed for each animal. The mean weight supported by the right hind paw was expressed relative to the total weight supported by both hind paws (right/(right + left)).

### Fibroblast-like synoviocyte cultures and treatment

Human fibroblast-like synoviocytes (FLS) were isolated from synovial membrane samples obtained from patients with osteoarthritis undergoing total hip replacement surgery (mean age: 75 years). Both male and female patients were included in the study. The experimental protocol was approved by the local ethics committee ("Comité de Protection des Personnes Nord-Ouest III", agreement #A13-D46-VOL.19). All participants provided written informed consent prior to surgery, and all procedures were conducted in accordance with applicable ethical guidelines and regulations.

Synovial membrane samples were enzymatically digested with collagenase type I (2 mg/mL, 12 h; Thermo Fisher Scientific, Waltham, USA) as previously described [24]. Released cells were cultured in high-glucose Dulbecco’s Modified Eagle Medium (DMEM) supplemented with glutamine and sodium pyruvate (Dutscher, Bernolsheim, France), 10% fetal bovine serum (FBS; Dutscher), and penicillin–streptomycin (Lonza, Basel, Switzerland), under standard conditions (37 °C, humidified atmosphere containing 5% CO₂). Cells were routinely tested for mycoplasma contamination by PCR.

For experiments, FLS between passages 3 and 8 were seeded at approximately 1 × 10^4^ cells/cm^2^. At confluence (approximately 5 days after seeding), recombinant human IL-1β was added at 1 ng/mL, either alone or together with EPZ-6438 (10 µM) and cells were maintained under these conditions for the indicated period.

### Immunocytochemistry and H3K27me3 staining

H3K27me3 staining was performed as previously described [7]. Briefly, fixed cells were incubated overnight with an anti-H3K27me3 antibody (C36B11, Cell Signaling Technology) followed by washing steps. An anti-rabbit IgG-FITC secondary antibody (sc-2012, Santa Cruz Biotechnology) was then applied, and nuclei were counterstained with DAPI. Fluorescence images were acquired using an EVOS FL Auto 2 Cell Imaging System (Thermo Fisher Scientific). H3K27me3 staining intensity was quantified from independent biological replicates.

### mRNA gene expression analysis

Total RNA was extracted from cells using the RNeasy Mini Kit (Qiagen, Hilden, Germany) according to the manufacturer's instructions. A DNase treatment step (AMPD1, Sigma-Aldrich) was performed to remove genomic DNA contamination. Reverse transcription was performed using 1 µg of total RNA and the M-MLV reverse transcriptase kit (Invitrogen, Carlsbad, USA). Quantitative real-time PCR was performed using the StepOne Plus Real-Time PCR System (Applied Biosystems, Courtaboeuf, France). Gene-specific primers were used at a final concentration of 200 nM in reactions containing Power SYBR Green Master Mix (Applied Biosystems). Relative gene expression levels were calculated using the comparative 2^−ΔΔCt^ method and normalized to the housekeeping gene RPL13.

### Autophagy assessment

Autophagy was evaluated using the Autophagy Assay Kit (MAK138, Sigma-Aldrich), which detects autophagosome formation through a fluorescent autophagosome marker (λex = 333 nm; λem = 518 nm). FLS were seeded at 10,000 cells/well in 96-well plates and exposed for 48 h to IL-1β (1 ng/mL), either alone or together with EPZ-6438 (10 µM). Cells were then incubated with the autophagy detection reagent for 30 min at 37 °C, washed according to the manufacturer's instructions, and fluorescence intensity was measured at excitation/emission wavelengths of 360/520 nm. Fluorescence images were acquired using identical acquisition settings within each independent experiment. Images were analyzed using Fiji (ImageJ), and the autophagy-associated fluorescent signal was quantified by image analysis and normalized to the total cell area.

### Proteomic experiments

Fibroblast-like synoviocytes derived from three independent osteoarthritis patients were cultured and treated with IL-1β (1 ng/mL) in the presence or absence of EPZ-6438 (10 µM). Cells were lysed and proteins were extracted using radioimmunoprecipitation assay (RIPA) buffer (50 mM Tris–HCl pH 7.5, 1% Igepal CA-630, 150 mM NaCl, 1 mM EGTA, 1 mM NaF, and 0.25% sodium deoxycholate) supplemented with protease and phosphatase inhibitor cocktails, as previously described [24]. Protein content was quantified by the Bradford method using a Bio-Rad assay (Marnes-la-Coquette, France). Samples were then submitted to the PROTEOGEN proteomics platform at the University of Caen Normandy for mass spectrometry analysis. Proteomic sample preparation and mass spectrometry acquisition were performed as previously described [26]. The mass spectrometer was operated in parallel accumulation–serial fragmentation (PASEF) mode. Ten PASEF MS/MS scans were acquired within a 1.25 s cycle time, with precursor ion charge states ranging from 2 to 5.

### Identification of differentially abundant proteins and enrichment analysis

Relative protein abundance was quantified using the label-free quantification (LFQ) workflow implemented in PEAKS X + software. Feature detection was performed independently for each sample using an expectation–maximization algorithm. Peptide features were aligned across replicates using retention time alignment algorithms. Mass error tolerance was set at 20 ppm, ion mobility tolerance at 0.07 (1/k0), and retention time tolerance at 10 min. Sample normalization was performed based on total ion current (TIC). Protein abundance was calculated using the summed intensity of the three most abundant unique peptides.

Differentially abundant proteins between experimental conditions were identified using paired Student’s t-test implemented in Perseus. Proteins displaying a fold change ≥ 1.2 and a p-value < 0.05 were considered significantly deregulated. Volcano plots were generated using Genoppi. Gene Ontology biological processes and Reactome pathway enrichment analyses were performed using STRING database version 12.0 [25]. Proteomic data are publicly available through the iProX integrated proteome resources database (dataset ID: IPX0011070000).

### ChiP-Seq

ChIP-seq experiments were performed by the Diagenode ChIP-seq/ChIP-qPCR Profiling Service using an anti-EZH2 antibody. ChIP-seq libraries were prepared and sequenced in paired-end mode (2 × 150 bp) on an Illumina NovaSeq 6000 platform using NovaSeq Control Software version 1.6.0. Sequencing quality was assessed using FastQC. Reads were aligned to the human reference genome (GRCh38/hg38) using BWA. Reads mapping to ENCODE blacklisted regions, multimapping reads, and PCR duplicates were removed using samtools. Alignment files were converted to BED format using BEDTools v2.17, and EZH2-enriched regions (peaks) were identified using MACS2. Peaks were visualized using the Integrative Genomics Viewer (IGV). Differential EZH2 binding profiles between experimental conditions were analyzed by identifying common and condition-specific peaks. Genes associated with condition-specific EZH2 promoter binding sites under IL-1β stimulation were subjected to Gene Ontology Biological Process enrichment analysis using STRING database (version 12.0). The ChIP-seq datasets generated in this study are available through the Gene Expression Omnibus database under accession number GSE300508.

### Macrophage differentiation and polarization

Macrophage differentiation and polarization of the THP-1 monocytic cell line (Merck, Sigma-Aldrich) were performed according to a protocol adapted from Baxter et al. [26]. THP-1 cells were maintained in RPMI medium supplemented with 10% fetal bovine serum and penicillin–streptomycin in a humidified atmosphere containing 5% CO₂ at 37 °C. Routine PCR-based testing was performed to monitor mycoplasma contamination.

For macrophage differentiation, THP-1 cells were seeded at 50 × 10^3^ cells/cm^2^ and stimulated with PMA (250 ng/mL) for 24 h. Cells were then washed and cultured for 72 h in PMA-free medium to obtain resting macrophages (M0-like macrophages). Pro-inflammatory polarization was induced by stimulation with IFNγ (20 ng/mL) and LPS from E. coli O55 (250 ng/mL) in the presence or absence of EPZ-6438 (10 µM).

Human bone marrow samples were collected from OA patients undergoing total hip replacement surgery (mean age = 70 years). The experimental protocol was approved by the local ethics committee, named "Comité de Protection des Personnes Nord Ouest III" (same agreement as for human synovium, #A13-D46-VOL.19). After red blood cell depletion using ACK lysing buffer, the resulting cell population was maintained for four days in DMEM containing 10% fetal bovine serum, penicillin–streptomycin, β-mercaptoethanol (50 µM). Cells were subsequently stimulated with M-CSF (50 ng/mL) and LPS (50 ng/mL) for two days in the presence or absence of EPZ-6438 (10 µM) to induce a pro-inflammatory macrophage phenotype. Each experiment was performed using independent biological samples from different OA patients.

### Flow cytometry

Spleens and lymph nodes (cervical, brachial, and inguinal lymph nodes) were dissociated through 40 µm cell strainers (Falcon). Cells were resuspended in staining buffer, and Fc receptors were blocked with anti-CD16/32 antibodies (10 µg/mL; clone 2.4G2, 553,142, BD Biosciences) at 4 °C for 15 min. Cells were then stained with fluorochrome-conjugated monoclonal antibodies for surface markers for 10 min in the dark at 4 °C. For intracellular staining, cells were fixed and permeabilized using the Inside Stain kit according to the manufacturer’s instructions (Miltenyi Biotec) before intracellular antibody staining.

The following antibodies were used for immune cell characterization: CD11b (REA592 VioBlue, 130–113–810, Miltenyi Biotec), F4/80 (REA126 PE-Vio770, 130–118–459, Miltenyi Biotec), CD80 (REA983 APC-Vio770, 130–116–463, Miltenyi Biotec), CD86 (REA1190 PE, 130–122–129, Miltenyi Biotec), MHC-II (REA813 FITC, 130–112–386, Miltenyi Biotec), CD38 (REA616 PerCP-Vio700, 130–109–260, Miltenyi Biotec), and iNOS (REA982 APC, 130–116–423, Miltenyi Biotec). Macrophages were identified based on CD11b and F4/80 expression, and inflammatory activation was assessed by the expression of CD80, CD86, MHC-II, CD38, and iNOS. Samples were acquired using a FACSVerse flow cytometer (BD Biosciences), and data were processed with FlowJo software version 7.6.5 (TreeStar Inc.).

### Osteoclastogenesis

Human bone marrow samples were collected from patients with osteoarthritis undergoing total hip replacement surgery (mean age = 73 years). After red blood cell depletion using ACK lysing buffer, cells were cultured at 300,000 cells/cm^2^ in α-MEM medium (Dutscher) supplemented with 10% fetal bovine serum, penicillin–streptomycin, β-mercaptoethanol (50 µM, Euromedex), and osteoclastogenic cytokines including RANK-L (30 ng/mL; 310–01, PreproTech) and M-CSF (25 ng/mL). Cells were cultured in the presence or absence of EPZ-6438 (10 µM). After approximately seven days, when multinucleated giant cells were observed, osteoclast differentiation was assessed by tartrate-resistant acid phosphatase (TRAP) staining according to the manufacturer’s protocol (387A, Sigma-Aldrich). TRAP-positive multinucleated cells were identified as osteoclasts and counted.

### Statistical analyses

All statistical procedures were conducted with GraphPad Prism 8 (GraphPad Software, San Diego, CA, USA). The Shapiro–Wilk test was used to evaluate the distribution of the data. Normally distributed continuous variables are presented as mean ± SEM and were analyzed using parametric tests, whereas non-normally distributed or ordinal data are presented as median with interquartile range (IQR) and were analyzed using non-parametric tests.

For in vitro experiments, paired statistical tests were used whenever matched biological samples were compared between experimental conditions. For in vivo experiments, comparisons between independent groups were performed using unpaired statistical tests. Two-group comparisons were analyzed using paired or unpaired Student's t-tests, or Mann–Whitney tests for non-parametric data, as appropriate. Multiple-group comparisons were performed using one-way or two-way ANOVA followed by Tukey's multiple comparisons test, or Kruskal–Wallis tests followed by Dunn's multiple comparisons test for non-parametric data, as appropriate. Outliers were identified using the ROUT method (Q = 1%).

For experiments using primary human cells, biological replicates corresponded to independent OA donors. For cell line experiments, biological replicates corresponded to independent experiments. Technical replicates were averaged and were not considered independent biological replicates for statistical analyses. Statistical significance was defined as p < 0.05.

## Supplementary Information

Supplementary Material 1.

## Data Availability

Data generated in this study are available from the NCBI Gene Expression Omnibus (GEO) (27) under accession number GSE300508 (https://www.ncbi.nlm.nih.gov/geo/query/acc.cgi?acc=GSE300508) or at iProX—integrated Proteome resources (Id: IPX0008212000).
