# Supplementary material for: EZH2 coordinates inflammatory and pain-associated pathways across joint compartments in osteoarthritis
Source: Mol Biomed. 2026 Oct 24;7:226. doi: 10.1186/s43556-026-00643-w (PMC13694607; doi:10.1186/s43556-026-00643-w)
Supplement: Supplementary file 1 [file 43556_2026_643_MOESM1_ESM.pdf]

EZH2 coordinates inflammatory and pain-associated pathways across joint compartments in osteoarthritis

Sybille BROCHARD[1], Alison VANLAEYS[1], Mahdia TAIEB[1], Isabelle RICHARD[1], Juliette AURY-LANDAS[1], Benoit BERNAY[2], Julien PONTIN[2], Celia SEILLIER[3], Nina BON[4], Baptiste PICART[1,5], Claire PECQUEUR[6], Olivier TOUTIRAIS[3], Claire VINATIER[4], Jérôme GUICHEUX[4], Eric MAUBERT[3], Véronique AGIN[3], Karim BOUMEDIENE[1], Catherine BAUGE[1]*

[1] Université de Caen Normandie, UR7451 BIOCONNECT, Caen, F-14000, France

[2] Université de Caen Normandie, US EMerode, PROTEOGEN, Caen, F-14000, France

[3] Normandie Univ, UNICAEN, INSERM, GIP Cyceron, Institut Blood and Brain @Caen-Normandie (BB@C), UMR-S U1237, Physiopathology and Imaging of Neurological Disorders (PhIND), Caen, F-14000, France

[4] Nantes Université, CHU Nantes, INSERM, Regenerative Medicine and Skeleton, RMeS, UMR 1229, Oniris, Nantes, F-44000, France

[5] Hôpital Privé St Martin, Service de Chirurgie Orthopédique, Caen, F-14000, France

[6] Nantes Université, Inserm 1307, CNRS 6075, Université d'Angers, CRCI2NA, F-44000 Nantes, France.

* Address for correspondence:

Catherine BAUGE

UR7451 BioConnecT

Université de Caen Normandie

14032 CAEN

FRANCE

Phone : +33 (0)2 31 06 82 73

E-mail : catherine.bauge@unicaen.fr

Table 1: List proteins deregulated by EPZ-6438 in IL-1 stimulated synoviocytes.

Accession	Gene name	Description	logFC	pvalue
P13667	PDIA4	Protein disulfide-isomerase A4	-3.40093	0.00160207
Q9P258	RCC2	Protein RCC2	-3.21243	0.0381512
P48735	IDHP	Isocitrate dehydrogenase [NADP] mitochondrial	-0.914807	0.00797848
Q9BZG1	RAB34	Ras-related protein Rab-34	-0.775697	0.0230256
P00568	KAD1	Adenylate kinase isoenzyme 1	0.203334	0.0340583
P36871	PGM1	Phosphoglucomutase-1	0.212835	0.0101563
P50395	GDIB	Rab GDP dissociation inhibitor beta	0.217763	0.0499717
P30044	PRDX5	Peroxiredoxin-5 mitochondrial	0.230621	0.0266481
Q96KP1	EXOC2	Exocyst complex component 2	0.238318	0.0483993
F5H365	F5H365	Protein transport protein SEC23	0.244969	0.0119121
P61106	RAB14	Ras-related protein Rab-14	0.267328	0.0245752
P18124	RL7	60S ribosomal protein L7	0.274041	0.0255669
O15173	PGRC2	Membrane-associated progesterone receptor component 2	0.283588	0.0305502
P40121	CAPG	Macrophage-capping protein	0.287716	0.0112755
P54578	UBP14	Ubiquitin carboxyl-terminal hydrolase 14	0.288739	0.0252215
Q8WUM4	PDC6I	Programmed cell death 6-interacting protein	0.289214	0.0382393
P49207	RL34	60S ribosomal protein L34	0.291148	0.0421039
P16152	CBR1	Carbonyl reductase [NADPH] 1	0.308976	0.0277092
P62979	RS27A	Ubiquitin-40S ribosomal protein S27a	0.310714	0.00684499
P20073	ANXA7	Annexin A7	0.316389	0.0237647
A0A0C4DGS1	A0A0C4DGS1	Dolichyl-diphosphooligosaccharide--protein glycosyltransferase 48 kDa subunit	0.318201	0.0475227
P50502	F10A1	Hsc70-interacting protein	0.342326	0.0170673
Q9Y285	SYFA	Phenylalanine--tRNA ligase alpha subunit	0.3437	0.0402616
Q53T59	H1BP3	HCLS1-binding protein 3	0.34453	0.0345412
P61020	RAB5B	Ras-related protein Rab-5B	0.345547	0.0144726
P18084	ITB5	Integrin beta-5	0.349897	0.0223674
Q5JR08	Q5JR08	Rho-related GTP-binding protein RhoC (Fragment)	0.350433	0.0467418
P52597	HNRPF	Heterogeneous nuclear ribonucleoprotein F	0.351871	0.00952878
P26640	SYVC	Valine--tRNA ligase	0.360656	0.0143028
O15118	NPC1	NPC intracellular cholesterol transporter 1	0.368992	0.020931
E9PLL6	E9PLL6	60S ribosomal protein L27a	0.369018	0.0198409
Q9H3U1	UN45A	Protein unc-45 homolog A	0.374484	0.0325441
P01023	A2MG	Alpha-2-macroglobulin	0.388073	0.0199562
P58335	ANTR2	Anthrax toxin receptor 2	0.402749	0.0458711
B8ZZQ6	B8ZZQ6	Prothymosin alpha	0.40537	0.0257504
A2A274	A2A274	Aconitate hydratase mitochondrial	0.419552	0.0157098
P32969	RL9	60S ribosomal protein L9	0.422249	0.00070824
P62826	RAN	GTP-binding nuclear protein Ran	0.429566	0.0358472
P07108	ACBP	Acyl-CoA-binding protein	0.429875	0.00098358
P52895	AK1C2	Aldo-keto reductase family 1 member C2	0.437757	0.0206759
O00499	BIN1	Myc box-dependent-interacting protein 1	0.438221	0.0301449
P30050	RL12	60S ribosomal protein L12	0.446763	0.0275765
O94832	MYO1D	Unconventional myosin-Id	0.454851	0.0292924
P04080	CYTB	Cystatin-B	0.462475	0.0247684
Q13442	HAP28	28 kDa heat- and acid-stable phosphoprotein	0.469984	0.0142197
P22695	QCR2	Cytochrome b-c1 complex subunit 2 mitochondrial	0.474312	0.0353184
Q14195	DPYL3	Dihydropyrimidinase-related protein 3	0.480645	0.0335528
Q16401	PSMD5	26S proteasome non-ATPase regulatory subunit 5	0.487153	0.0105066
O95202	LETM1	Mitochondrial proton/calcium exchanger protein	0.48747	0.033909
Q9UHV9	PFD2	Prefoldin subunit 2	0.491241	0.0287517
A0A0A6YYA0	A0A0A6YYA0	Protein TMED7-TICAM2	0.491629	0.0328677
E7EPK1	E7EPK1	Septin	0.494426	0.038302
A0A1W2PQT3	A0A1W2PQT3	Malic enzyme	0.498047	0.00788401
P32321	DCTD	Deoxycytidylate deaminase	0.502155	0.0411568
P61923	COPZ1	Coatomer subunit zeta-1	0.532699	0.0439205
A0A0A0MS51	A0A0A0MS51	Actin-depolymerizing factor	0.533591	0.0391932
Q00839	HNRPU	Heterogeneous nuclear ribonucleoprotein U	0.533669	0.031206
P09497	CLCB	Clathrin light chain B	0.536462	0.0376439
P08253	MMP2	72 kDa type IV collagenase	0.539859	0.0159
P07204	TRBM	Thrombomodulin	0.544198	0.0312993
Q15046	SYK	Lysine--tRNA ligase	0.544365	0.0284348
A0A1C7CYX9	A0A1C7CYX9	Dihydropyrimidinase-related protein 2	0.545378	0.00983617
P17931	LEG3	Galectin-3	0.548639	0.0268366
Q15717	ELAV1	ELAV-like protein 1	0.553402	0.00215299

Q99536	VAT1	Synaptic vesicle membrane protein VAT-1 homolog	0.561105	0.0390699
P11766	ADHX	Alcohol dehydrogenase class-3	0.56493	0.0103023
Q9NX46	ADPRS	ADP-ribose glycohydrolase ARH3	0.573112	0.0420053
P55036	PSMD4	26S proteasome non-ATPase regulatory subunit 4	0.59972	0.0498809
P27105	STOM	Stomatin	0.618933	0.0477259
O95295	SNAPN	SNARE-associated protein Snapin	0.624175	0.0355106
P07900	HS90A	Heat shock protein HSP 90-alpha	0.63659	0.00174499
Q9NZU5	LMCD1	LIM and cysteine-rich domains protein 1	0.636793	0.0483049
P20742	PZP	Pregnancy zone protein	0.654117	0.0137395
Q14444	CAPR1	Caprin-1	0.686128	0.0423988
P60866	RS20	40S ribosomal protein S20	0.68851	0.0207338
J3QRI7	J3QRI7	60S ribosomal protein L26 (Fragment)	0.698098	0.0417818
P42677	RS27	40S ribosomal protein S27	0.72299	0.0350072
P12268	IMDH2	Inosine-5'-monophosphate dehydrogenase 2	0.741286	0.0447653
P62829	RL23	60S ribosomal protein L23	0.742774	0.0441349
A0A7P0Z4A2	A0A7P0Z4A2	Sorting nexin-9	0.755497	0.0424821
Q8N5K1	CISD2	CDGSH iron-sulfur domain-containing protein 2	0.767913	0.00676764
Q13263	TIF1B	Transcription intermediary factor 1-beta	0.769998	0.0401198
P48426	PI42A	Phosphatidylinositol 5-phosphate 4-kinase type-2 alpha	0.770961	0.0340927
A0A087WY55	A0A087WY55	Chromosome 6 open reading frame 55 isoform CRA_b	0.772326	0.0324466
Q8WWM9	CYGB	Cytoglobin	0.774351	0.0109495
A0A2R8Y484	A0A2R8Y484	Integrin-associated protein (Fragment)	0.774624	0.0282902
Q9Y2A7	NCKP1	Nck-associated protein 1	0.779765	0.0488668
P41226	UBA7	Ubiquitin-like modifier-activating enzyme 7	0.789098	0.0454478
E9PNW4	E9PNW4	CD59 glycoprotein	0.789828	0.0113317
E5RGS4	E5RGS4	Prefoldin subunit 1	0.79671	0.0163729
Q9NT62	ATG3	Ubiquitin-like-conjugating enzyme ATG3	0.800326	0.0452554
Q9BVC6	TM109	Transmembrane protein 109	0.805061	0.00516605
E9PH64	E9PH64	NADH dehydrogenase [ubiquinone] 1 beta subcomplex subunit 9	0.807782	0.0439846
P23381	SYWC	Tryptophan--tRNA ligase cytoplasmic	0.812357	0.0415376
A0A2R8Y7R2	A0A2R8Y7R2	Hemoglobin subunit beta	0.861646	0.00354612
P36405	ARL3	ADP-ribosylation factor-like protein 3	0.890901	0.0289507
Q9Y265	RUVB1	RuvB-like 1	0.895786	0.0269516
P46976	GLYG	Glycogenin-1	0.907195	0.0316347
P55735	SEC13	Protein SEC13 homolog	0.935264	0.0292658
P80723	BASP1	Brain acid soluble protein 1	0.95867	0.0193001
P61201	CSN2	COP9 signalosome complex subunit 2	0.984665	0.0150697
Q2TAA2	IAH1	Isoamyl acetate-hydrolyzing esterase 1 homolog	1.0192	0.00271183
Q8NDC0	MISSL	MAPK-interacting and spindle-stabilizing protein-like	1.02822	0.0419808
Q96AT9	RPE	Ribulose-phosphate 3-epimerase	1.03937	0.0471483
D6RFH4	D6RFH4	Cytochrome b5 type B	1.07564	0.0476349
P50281	MMP14	Matrix metalloproteinase-14	1.10562	0.0462285
Q5T7C4	Q5T7C4	High mobility group protein B1	1.10696	0.044598
P02795	MT2	Metallothionein-2	1.1112	0.0253598
Q9BPX5	ARP5L	Actin-related protein 2/3 complex subunit 5-like protein	1.16067	0.00224028
Q9GZT8	NIF3L	NIF3-like protein 1	1.16915	0.00787084
A0A0C4DGV4	A0A0C4DGV4	Late endosomal/lysosomal adaptor and MAPK and MTOR activator 5	1.18292	0.0463546
A0A3B3IRS7	A0A3B3IRS7	Lysosomal cobalamin transport escort protein LMBD1	1.22317	0.0130033
Q5H9A7	Q5H9A7	Metalloproteinase inhibitor 1	1.22839	0.0209772
P21817	RYR1	Ryanodine receptor 1	1.2339	0.0305715
P48643	TCPE	T-complex protein 1 subunit epsilon	1.26143	0.0342727
Q6IBS0	TWF2	Twinfilin-2	1.28027	0.0435127
P09619	PGFRB	Platelet-derived growth factor receptor beta	1.3079	0.0286426
P51151	RAB9A	Ras-related protein Rab-9A	1.32109	0.0429648
P02545	LMNA	Prelamin-A/C	1.44821	0.0455913
Q00653	NFKB2	Nuclear factor NF-kappa-B p100 subunit	1.45501	0.00848168
P17813	EGLN	Endoglin	1.52678	0.0353231
O60547	GMDS	GDP-mannose 4 6 dehydratase	1.54079	0.0320901
Q14165	MLEC	Malectin	1.5523	0.0359763
A0A7I2V5M3	A0A7I2V5M3	Procathepsin L	1.68857	0.0410056
Q07955	SRSF1	Serine/arginine-rich splicing factor 1	1.84574	0.0347603
P69905	HBA	Hemoglobin subunit alpha	2.06381	0.0288423
O94874	UFL1	E3 UFM1-protein ligase 1	2.29529	0.00484066
P62070	RRAS2	Ras-related protein R-Ras2	2.34482	0.0169834

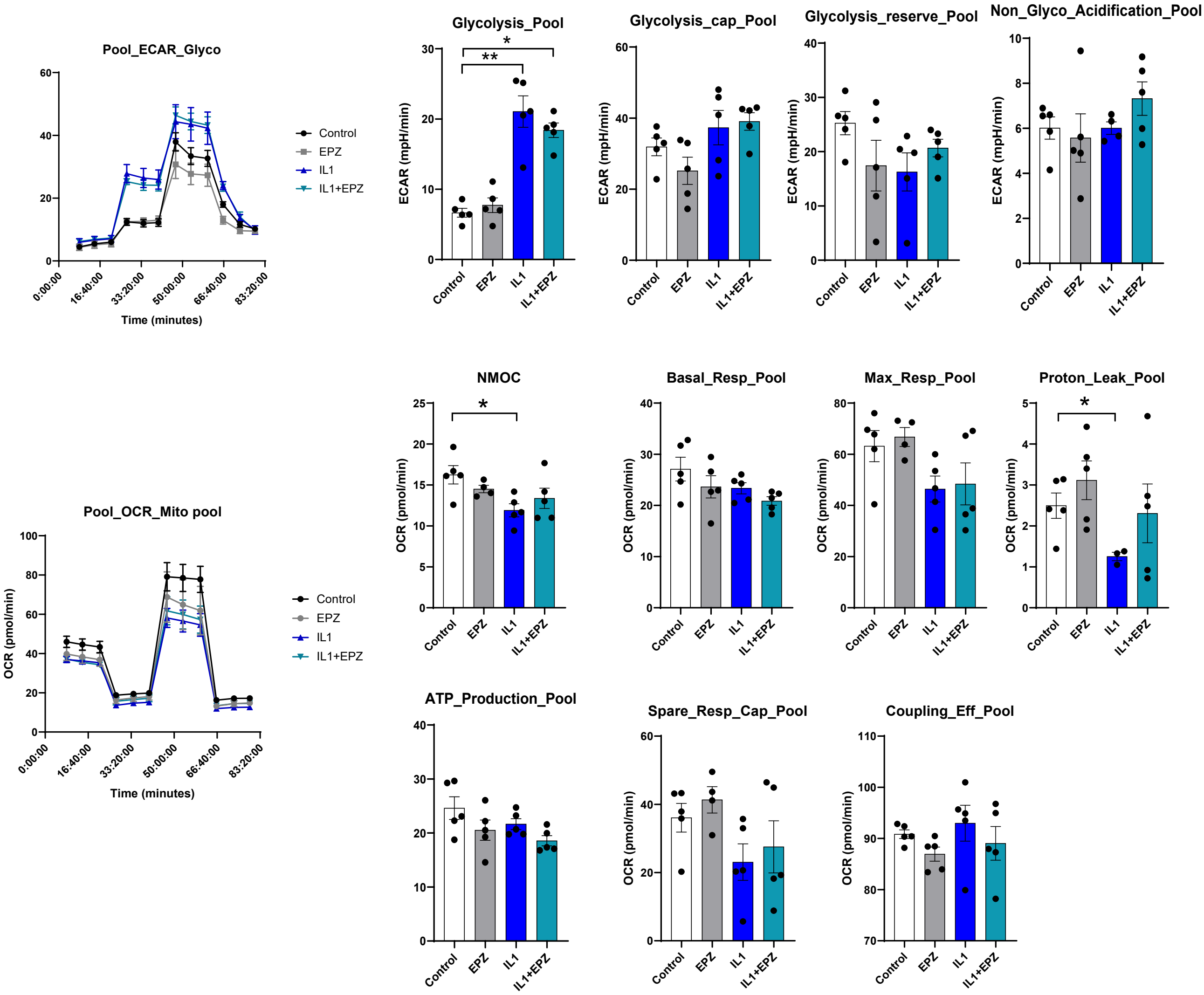

Figure S1: Seahorse analysis of metabolic parameters following EZH2 inhibition in OA fibroblast-like synoviocytes.

Fibroblast-like synoviocytes were stimulated with IL-1β (1 ng/ml) in the presence or absence of EPZ-6438 (10 μM). After 24 h of treatment, glycolytic and mitochondrial activities were assessed by Seahorse extracellular flux analysis. Extracellular acidification rate (ECAR) and oxygen consumption rate (OCR) were measured every 5 minutes before and after sequential addition of metabolic modulators. Data were analyzed using Wave software. Experiments were independently repeated at least three times. Data are presented as mean ± SEM. Statistical significance was defined as *$p < 0.05$ and **$p < 0.01$.

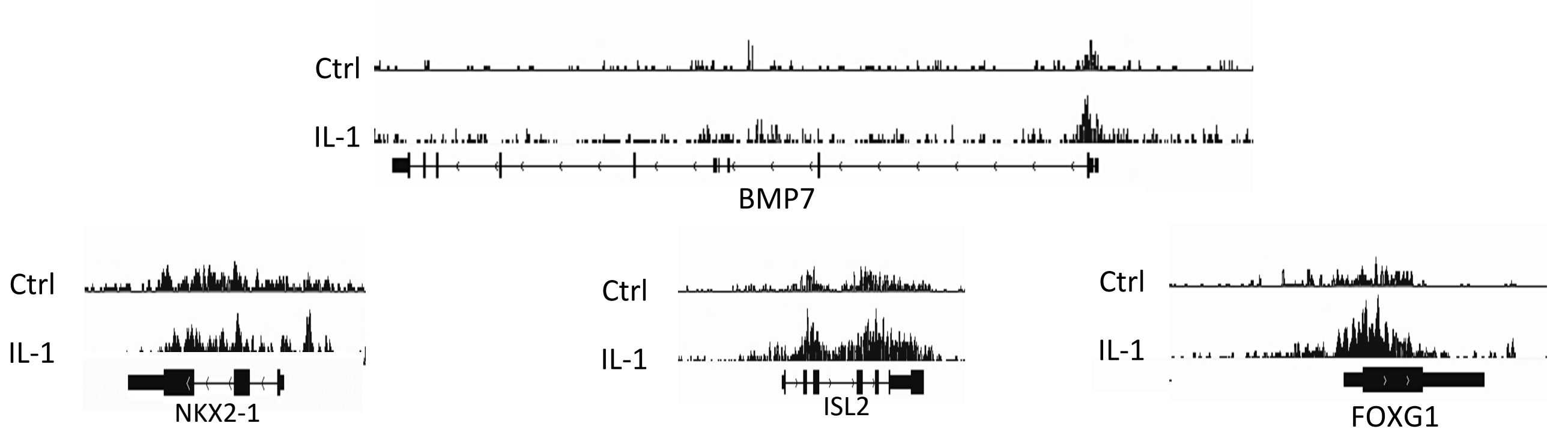

Figure S2: EZH2 occupancy at promoters of genes associated with neurogenesis and axon guidance pathways. Fibroblast-like synoviocytes were stimulated with IL-1β for 48 h, and EZH2 chromatin occupancy was assessed by ChIP-seq analysis (as described in Figure 5). A binary comparison of ChIP-seq peaks was performed to identify common and condition-specific EZH2-bound regions. Representative EZH2-enriched peaks located at promoter regions of BMP7, NKX2-1, ISL2, and FOXG1, genes associated with neurogenesis and axon guidance processes, are shown.

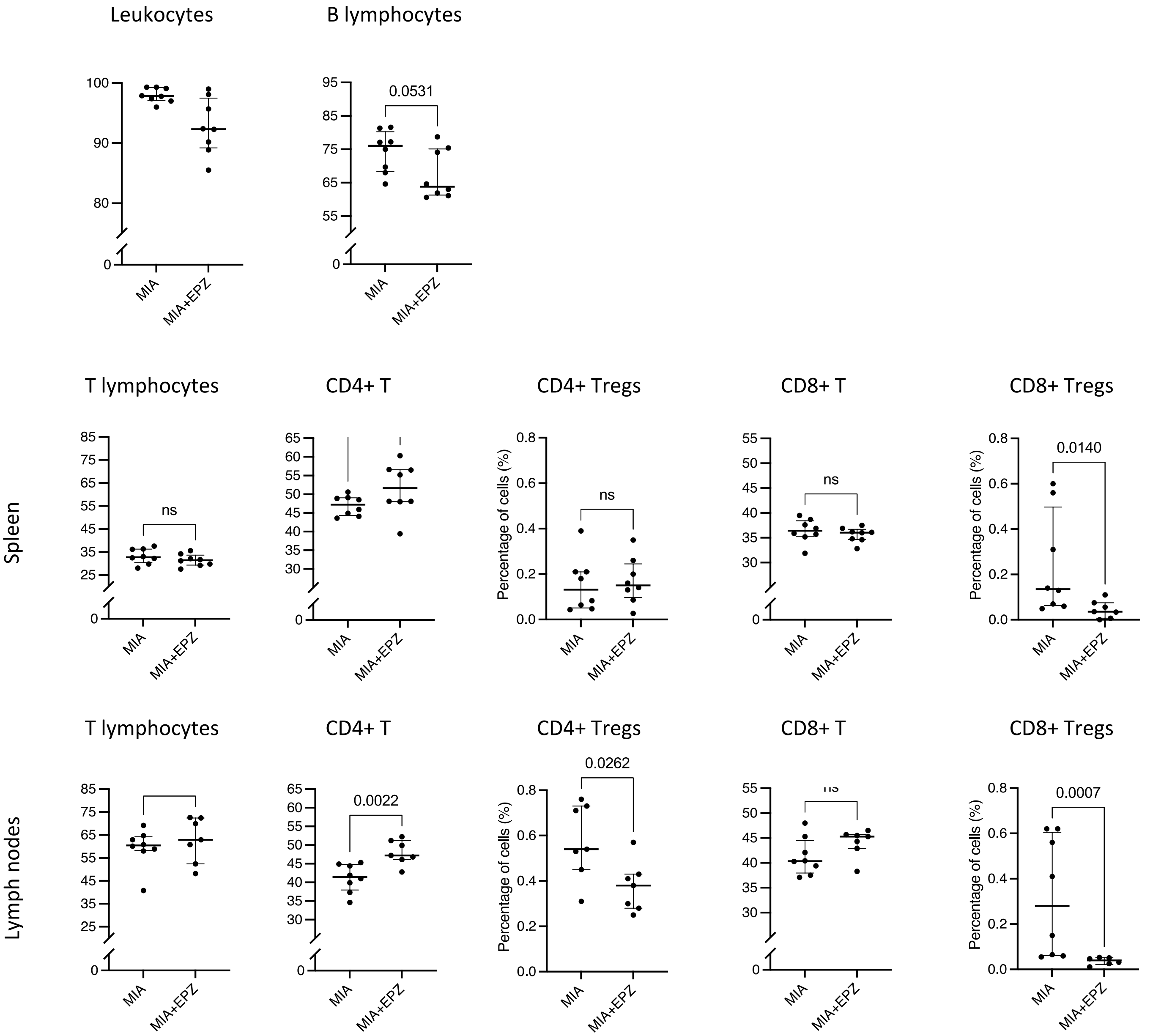

Figure S3: Flow cytometric characterization of lymphocyte populations in spleen and lymph nodes of OA mice following EZH2 inhibition.

Leukocyte and lymphocyte subsets were analyzed by flow cytometry in spleen and lymph nodes from OA mice treated with vehicle or EPZ-6438. (a) Percentage of total leukocytes and B lymphocytes in the spleen. (b) Frequency of total T lymphocytes, $CD4^+$ T cells, $CD4^+$ regulatory T cells (Tregs), $CD8^+$ T cells, and $CD8^+$ Tregs in the spleen. (c) Frequency of total T lymphocytes, $CD4^+$ T cells, $CD4^+$ Tregs, $CD8^+$ T cells, and $CD8^+$ Tregs in lymph nodes. Cells were isolated and stained with appropriate surface markers for flow cytometry analysis. Samples were acquired using a FACSVerse cytometer and analyzed with FlowJo software. Results are presented as median ± interquartile range (n = 8 mice). Outliers were identified using the ROUT method (Q = 1%) and may have been excluded, potentially resulting in different numbers of analyzed samples. Each dot represents an individual mouse. Statistical significance was assessed using the Mann–Whitney test; $p < 0.05$ was considered statistically significant.
